# The flip side of frequent sanitising and hand washing

**DOI:** 10.4102/safp.v65i1.5595

**Published:** 2023-01-11

**Authors:** Lehlohonolo Makhakhe

**Affiliations:** 1 The South African Institute of Dermatology, Bloemfontein, South Africa; 2 Department of Dermatology, Faculty of Health Science, University of the Free State, Bloemfontein, South Africa

**Keywords:** hand washing, xerosis, sanitiser, contact dermatitis, COVID-19

## Abstract

The stratum corneum is the outermost layer of the epidermis. It acts as an interface with the external environment and functions as a barrier that prevents microorganisms and allergens from penetrating the skin, while preventing bodily fluids, electrolytes and proteins from being lost in a process aimed at maintaining homeostasis. With the novel coronavirus disease 2019 (COVID-19) outbreak, there has been an increase in hygiene practice, particularly hand washing and the use of hand sanitisers. These practices have undoubtedly assisted a great deal in combatting the rate of transmission and contributed immensely to saving lives. However, repeated hand washing and the use of sanitisers have both been linked with marked skin dryness and contact dermatitis. This especially holds true when the above-mentioned practices are carried out in the absence of intermittent hand moisturiser usage.

## Introduction

Since the start of the coronavirus disease 2019 (COVID-19) global outbreak back in late 2019, medical experts through mainstream media and publications have consistently advocated for social distancing, wearing of masks, sanitising and regular hand washing as effective methods of combatting the pandemic.^1^

Such practical measures have assisted tremendously in saving lives and reducing the transmission of the virus beyond the millions of confirmed cases around the globe.^2^

According to the World Health Organization (WHO), good hand hygiene and other cost-effective infection prevention and control (IPC) practices can eliminate between 35% and 70% of healthcare-setting infections in all countries regardless of economic status.^3^

Despite the pandemic, high-income countries were eight times more likely to implement advanced IPC than middle and low-income countries.^3^

## Background

The recent COVID-19 pandemic has resulted in worldwide awareness of hand hygiene and hand cleansing. Hand hygiene is a widely accepted principle in the prevention of disease transmission because proper hand hygiene has a 24% to 31% likelihood of decreasing the spread of transmissible disease.^4^

Hand hygiene products are available in a variety of forms, and while each of these formulations may be effective against COVID-19, they may also alter skin barrier integrity and function. Alcohol-based hand sanitisers with moisturisers have the least sensitising and irritancy potential when compared to soaps and synthetic detergents.^4,5^

As an additional measure to social distancing and all the recommended measures by authorities, most healthcare workers and the general public became engaged in repetitive and aggressive hand sanitising and hand washing practice.

**FIGURE 1:**
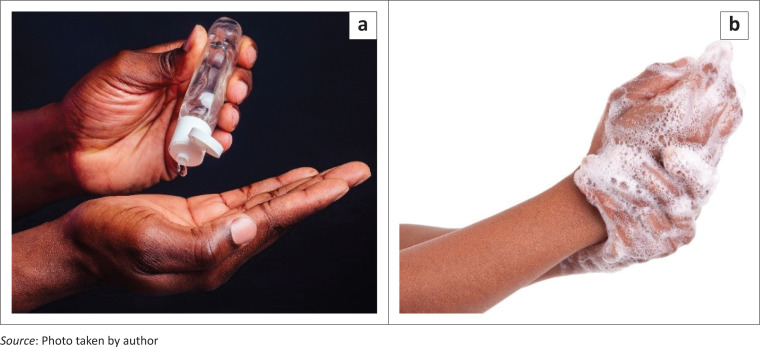
The regular use of sanitizers and frequent hand washing practice.

Sanitising, hand washing and the frequent use of soaps remain effective in killing the novel coronavirus and thus delay the spread (Figure 1).

However, research has shown that not many people use emollients congruent with the increased sanitising and copious hand washing practices.^5,6^

**FIGURE 2:**
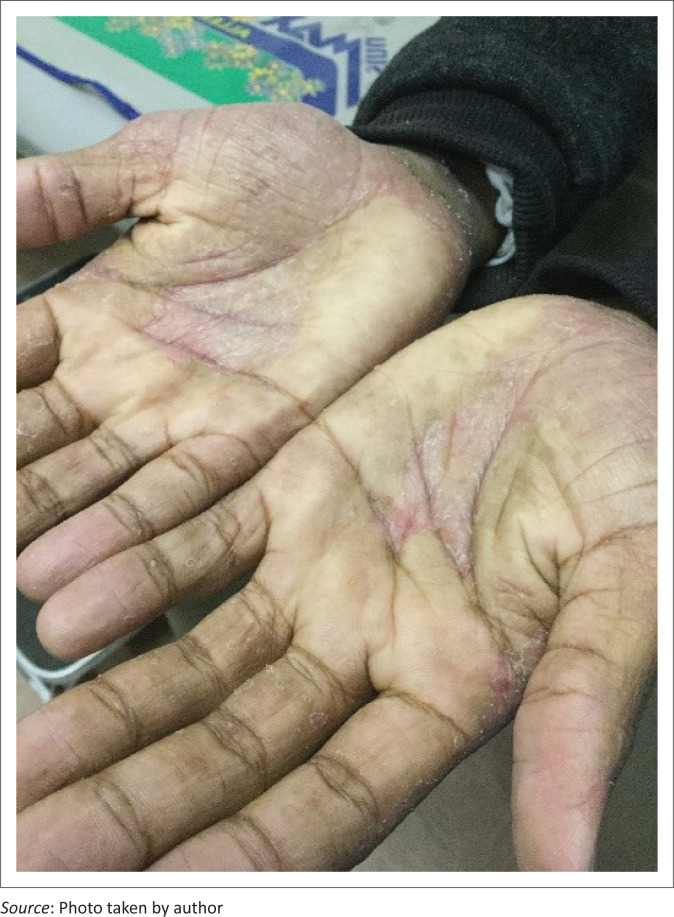
Chronic irritant contact dermatitis secondary to the usage of sanitisers, affecting both hands on the palmar surface.

Hand washing, especially with hot water has been directly linked to skin dryness (xerosis), through transcutaneous water loss (see Figure 2).^6^

The use of sanitisers, especially the alcohol-based types, is strongly associated with skin moisture loss and irritant contact dermatitis due to their harshness on the skin. This is due to the negative effect that alcohol-based sanitisers have on skin lipids and fats that form part of the external skin structure and barrier.^7^

Due to their high pH, preservatives and colourants content, skin irritation and dermatitis were noted as a result of overzealous hand soap washing that was frequent and often times prolonged with no moisturisers used afterwards to repair the barrier properties of the skin.

Both bar and liquid hand soaps are commonly used as cleansers. They typically have an alkaline pH of 9–10, because of their prototypical anionic surfactant nature, their use can result in marked skin dryness and accompanied irritation. In a study, the general prevalence rate of occupational skin damages was 97.0% (526/542) among first-line healthcare workers with the hands being significantly affected.^8^

The skin is the first line of defence and barrier against different types of infections. Its integrity can be lost due to various external factors, leading to loss of moisture, dryness and pruritus.^9^

The protective nature of the skin allows for a physiological shield that protects against the entry of allergens, microbes, including viruses of different types. The same barrier helps in maintaining homeostasis, by preventing loss of body fluid and electrolytes. Compromise in the barrier can result in invasion of foreign bodies transcutaneously, introduction of toxins into the deeper skin structures as well as loss of fluids through interrupted skin integrity. Loss of fluids in turn leads to skin dryness and subsequent itching.^10^

Intensified handwashing and disinfection can cause irritant contact dermatitis by interrupting the epidermal barrier functions. As in other areas of occupational dermatology, the use of disinfectants alone is better tolerated than the combination of soap and disinfectant. Protective creams and other forms of emollients before the shift and moisturisers during the day reduce the risk of occupational hand dermatitis.

Soap removes dirt and inactivates viruses by disrupting the lipid membrane and intracellular lipids. There is evidence to support soap as a more effective method of hand hygiene than hand sanitiser.

**FIGURE 3:**
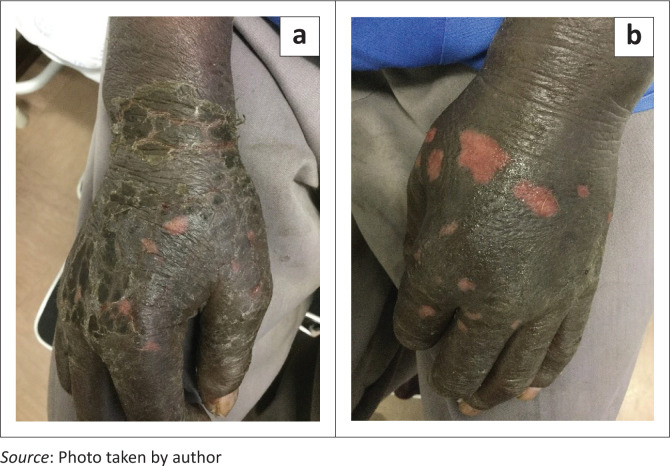
Sub-acute contact dermatitis secondary to the usage of latex gloves, characterised by erosions, swelling and scaling.

Latex glove usage by those combating the pandemic as front-line healthcare workers also resulted in increased incidence of hand dermatitis. This led to symptoms such as local itching and occasional pain. Clinical signs noted were swelling, diffuse erythema, pustulation, vesiculation, scaling and pigment changes both on the dorsal and palmar aspects of the hands (Figure 3).^11^

Other clinical signs noted often are xerosis, eczematous lesions, and even maceration. Despite the increased frequency of handwashing and disinfection, a recently conducted survey revealed that only 22.1% used moisturisers regularly.^9^

## Management

### Non-pharmacological advice (prevention)

Avoidance of identified triggers, depending on the ingredients in some soaps and sanitisers, not all result in skin reactions, even in sensitive skin type of individuals.

The use of hand moisturisers after hand washing and regularly during the day is highly recommended.

For sensitive skin, emollients that are hypo-allergic, preservative-free and oil in cream-based type of formulation is advised to reduce the risk of allergies.

Soap subsets that are hypo-allergic will also result in lesser skin irritation, while removing unwanted microorganism and exfoliated (dead) corneum cells.

Other soap subsets that are of unquestionable importance include glycerin and superfatted soaps. They contain humectants, which help significantly in retaining moisture after hand washing.

Lukewarm water is advised over the use of hot water in hand washing. This prevents moisture loss as a result of skin dehydration.

With any noticeable skin changes that are causing consternation or medical concern, a dermatologist must be consulted.

### Pharmacological considerations

- Hand creams and other types of emollients: These are mostly over-the-counter products, highly effective in dry skin and restoring the skin barrier.
- Analgesics: In cases of pain or tenderness associated with the skin reaction, mostly with contact dermatitis.
- Oral or topical antihistamines: Can be prescribed when skin reactions are associated with marked itching for a specified duration based on clinical exam, the degree and extent of involvement.
- Low to medium potency corticosteroids: In cases of dermatitis, steroids assist as anti-inflammatory agents through action on lymphocytes and other immune regulating cells.^12^
- Oral corticosteroids: Highly effective in acute management of most forms of dermatitis.

## Conclusion

The use of alcohol-based sanitisers and regular hand washing remains key in good hygiene practice and reducing the spread of all forms of microbes. However, the dangers of sanitisers and frequent hand washing also need to be emphasised. Regular usage of emollients like hand creams remains key in reducing the unwanted drying effects of repeated hand washing and the potential allergies linked to strong sanitiser usage.
